# Dynamic Correlations and Disorder in the Masticatory Musculature Network

**DOI:** 10.3390/life13112107

**Published:** 2023-10-24

**Authors:** Gaetano Campi, Alessandro Ricci, Nicola Costa, Federico Genovesi, Jacopo Junio Valerio Branca, Ferdinando Paternostro, Daniele Della Posta

**Affiliations:** 1 Institute of Crystallography, CNR, Via Salaria Km 29.300, 00015 Monterotondo, Italy; gaetano.campi@ic.cnr.it; 2 Duferco Corporate Innovation, Via Trevano 2A, 6900 Lugano, Switzerland; phd.alessandro.ricci@gmail.com; 3 The Anatomical Network APS, Via Fermo 2c, 00182 Rome, Italy; nicolacosta.osteopatia@gmail.com (N.C.); osteodan@gmail.com (D.D.P.); 4 Manchester City Football Club, Manchester M11 3FF, UK; genovesifederico5@gmail.com; 5 Department of Experimental and Clinical Medicine, Anatomy and Histology Section, University of Florence, 50134 Florence, Italy; jacopojuniovalerio.branca@unifi.it

**Keywords:** temporomandibular joint disorder, graph theory, dynamic correlations, stable distributions

## Abstract

Background: Temporomandibular joint (TMJ) disorders, which affect millions of people worldwide, have multiple etiological factors that make an accurate diagnosis and effective treatments difficult. As a consequence, the gold standard diagnostic criteria for TMJ disorders remain elusive and often depend on subjective decisions. Aim: In this context, the lack of a non-invasive quantitative methodology capable of assessing the functional physiological state and, consequently, identifying risk indicators for the early diagnosis of TMJ disorders must be tackled and resolved. Methodology: In this work, we have studied the biomechanics and viscoelastic properties of the functional masticatory system by a non-invasive approach involving 52 healthy subjects, analysed by statistical–physics analysis applied to myotonic measurements on specific points of the masticatory system designing a TMJ network composed of 17 nodes and 20 links. Results: We find that the muscle tone and viscoelasticity of a specific cycle linking frontal, temporal, and mandibular nodes of the network play a prominent role in the physiological functionality of the system. At the same time, the functional state is characterised by a landscape of nearly degenerated levels of elasticity in all links of the network, making this parameter critically distributed and deviating from normal behaviour. Conclusions: Time evolution and dynamic correlations between biomechanics and viscoelastic parameters measured on the different cycles of the network provide a quantitative framework associated with the functional state of the masticatory system. Our results are expected to contribute to enriching the taxonomy of this system, primarily based on clinical observations, patient symptoms, and expert consensus.

## 1. Introduction

The masticatory system is one of the most important modules in the human anatomical network [1]. It is composed of functional tissues and organs whose main tasks are food intake, the initial stage of mastication, and digestion. Beyond these main tasks, the system is also involved in several important aspects, particularly with a high impact on both physiological and psychological processes, such as emotional expression, speech articulation, breathing, and body balance [2,3]. In this context, a functional disorder of the masticatory system can affect various aspects of health and can manifest as muscle pain, crackling of the temporomandibular joints (TMJs), impaired mobility, clenching of teeth, or hearing damage. Early studies in this field concerning the influence of dental occlusion on muscular activity were performed by Hickey et al. in the 1950s [4]. Functional disorders of the masticatory system can also affect speech sounds [5]. Indeed, the masticatory system and speech-sound disorders are found together in many dental, orthodontic, laryngological, phonetic, and speech therapy studies. Evidence to date indicates a deep interrelationship of jaw, neck, and trunk muscle activity [6], and it seems that neck muscle contraction is tuned with the mandibular muscles during mandibular activity [7]. Furthermore, recent research works have established that masticatory function plays a relevant role in the development of cognitive activity and decay [8].

## 2. Objectives and Aims

The taxonomy of the temporomandibular Joint (TMJ) is primarily based on clinical observations, patient symptoms, and expert consensus, lacking a physical–mathematical model as a reference. In the realm of muscle functionality and its mechanical properties, quantitative investigative techniques encompass electromyography (EMG) [9] and ultrasonography (USG) [10]. Ultrasonic imaging comprises two distinct modalities: strain and shear wave elastography [11]. Strain elastography relies on the application of external pressure to tissues, generating a quantitative stiffness map based on relative distortion imaging [12]. Nevertheless, this technique is not without limitations, notably in terms of the potential for object displacement due to applied pressure. To mitigate these issues, shear wave elastography (SWE) emerged [11]. SWE is a non-invasive method characterised by ultrafast processes capable of ascertaining the elasticity of diverse tissues [13,14,15,16] through the calculation of shear wave propagation speeds within the tissues [17,18]. In recent developments, non-invasive portable hand-held devices for myotonometry have been employed to objectively evaluate muscle spasticity by quantifying tissue displacement in response to perpendicular compression forces [16,19]. It is noteworthy that the myotonometric measurements obtained through EMG and USG exhibit strong correlations with the measurements obtained using the MyotonPRO device [20]. The MyotonPRO is a relatively new portable handheld apparatus that offers a simple and non-invasive means to characterise the tone, mechanical stiffness, and viscoelasticity of skeletal muscle [21,22]. Its operations entail the application of a mechanical impulse to the skin, which is subsequently transmitted to the underlying soft tissue and muscle. This impulse provokes a damped natural oscillation in the muscle, recorded by an accelerometer in the form of an accelerogram [23]. Hence, within this work, we have applied a statistical–physics approach to MytonPRO measurements, seeking to establish a quantitative framework of the biomechanical and viscoelastic properties of the masticatory system in its functional state.

The primary objective of this study is to probe masticatory function by conducting an exhaustive analysis of the anatomical network encompassing the muscles in the neck and head region, including those involved in the process of chewing. This analysis aims to unveil the intricate interactions among anatomical components implicated in chewing, providing a holistic perspective on the coordination and function of these components. The transition from a functional to a pathological state is frequently gradual, rendering a clear demarcation between the two somewhat challenging. Therefore, the second significant objective of this research study is to introduce the myotonometer (MyotonPRO) as an innovative tool in the domain of functional masticatory diagnostics. This device is designed to precisely gauge muscle tension and the elasticity of muscle tissues within the anatomical network. The joint utilization of the anatomical network model and the myotonometer represents an advanced approach to obtaining a comprehensive and detailed assessment of muscle function, with a specific focus on patients afflicted by temporomandibular disorders (TMDs) or other chewing-related conditions.

Indeed, the development of symptomatic pathological states depends on various subjective factors, encompassing tissue reactivity as well as the patient’s personality and physical condition. Irrespective of this pre-existing knowledge, a comprehensive understanding of the physiological state is imperative for the detection of deviations, facilitating the identification of the initial phase of pathology onset. For example, an intrinsic factor contributing to physiological degeneration within the masticatory system is the aging process [24,25,26,27].

This foundational knowledge serves as a launchpad for future research endeavours focused on TMD patients. The objective is to pinpoint potential irregularities or disparities within the anatomical network and assess their potential linkage to chewing disorders. The enhancement of this integrated approach can significantly advance the assessment and management of TMD patients, contributing to the improvement of quality in care offered to those grappling with these disorders, helping monitor patients during a rehabilitation phase of masticatory function [28], and, furthermore, intercepting a misknown masticatory dysfunction in healthy subjects.

## 3. Materials and Methods

The current work is a cohort study that provides statistical data derived from measurements of muscle tone of the masticatory system conducted on 52 individuals out of a total of 163 voluntary participants. At the time of the measurements, the volunteers ages ranging from 20 to 86 years included males (*n* = 29) and females (*n* = 23). The participants were selected based on the exclusion criteria, which ensured the absence of any temporomandibular disorders, whether articular, muscular, or any other symptoms related to the masticatory system [29]. Furthermore, none of these individuals received dental care during the period in which the measurements were taken or in the 12 months leading up to them.

The participants consented to participate in this study. Measurements were taken at rest on both sides of the individuals’ faces on sets of five muscles with masticatory functionality, some directly implicated in masticatory functionality and others involved in head and neck movement, which is often associated with and perturbed by masticatory function [30,31,32].

**Figure 1:**
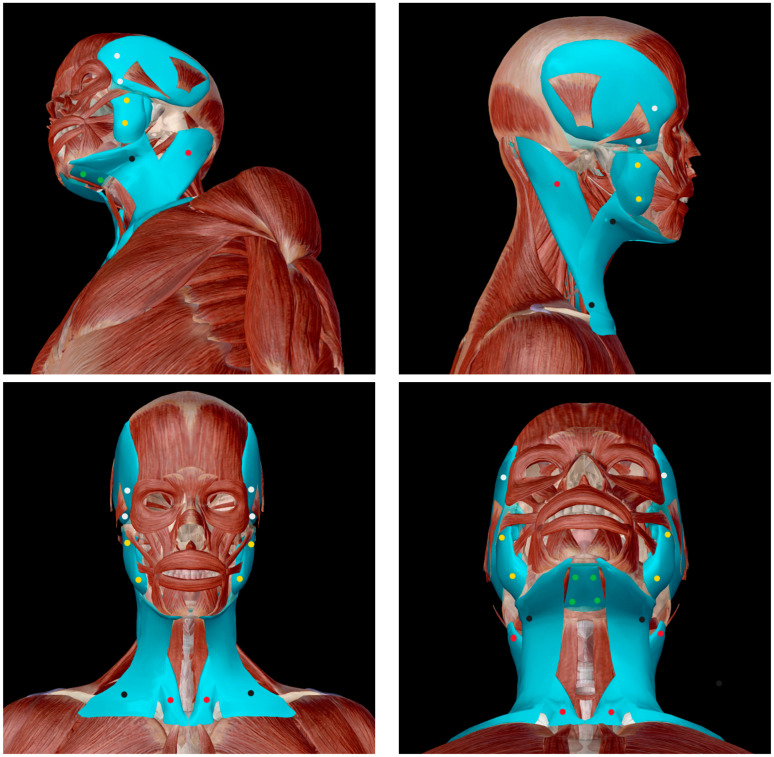
Muscle taken into consideration for measurement with the use of MyotonPRO. The coloured points show the 20 points where measurements have been taken by MyotonPRO on each subject. The legend describes the five pairs (right (RT) and left (LT)) of tested muscles, divided by colour, and their corresponding bony insertions, (www.visiblebody.com) as reported in the following list: M. temporal RT (interacts with) frontal (white); m. temporal RT (interacts with) mandible (white); m. masseter RT (interacts with) temporal (yellow); m. masseter RT (interacts with) mandible (yellow); m. mylohyoid RT (interacts with) mandible (green); m. mylohyoid RT (interacts with) hyoid bone (green); m. platysma RT (interacts with) mandible (black); m. platysma RT (interacts with) clavicle RT (black); m. sternocleidomastoid RT (interacts with) clavicle RT (red); m. sternocleidomastoid RT (interacts with) temporal (red); m. temporal LT (interacts with) frontal (white); m. temporal LT (interacts with) mandible (white); m. masseter LT (interacts with) temporal (yellow); m. masseter LT (interacts with) mandible (yellow); m. mylohyoid LT (interacts with) mandible (green); m. mylohyoid LT (interacts with) hyoid bone (green); m. platysma LT (interacts with) mandible (black); m. platysma LT (interacts with) clavicle (black); m. sternocleidomastoid LT (interacts with) clavicle (red); m. sternocleidomastoid (interacts with) temporal (red).

The measurements were carried out in specific areas to standardise the procedure concerning the bony insertion of temporalis, masseter, mylohyoid, platysma, and sternocleidomastoid muscles. As reported and detailed in Figure 1, 20 points were taken into consideration, corresponding to the muscle areas near their bony insertions.

The MyotonPRO instrument measures the tone, the biomechanical, and viscoelastic response of the muscle to a brief (15 ms) mechanical impulse (with a force of 0.58 N) on the skin surface above the muscle [26]. The mechanical deformation of the tissue is delivered by the device testing end held perpendicular to the skin surface. An integrated 3-axis digital acceleration sensor recorded the muscle dynamic response, providing an accelerogram composed of damped oscillations. The analysis of the recorded signal is performed in real time by an integrated software allowing the extraction of the following five parameters, namely *A^m^*: frequency, F (*m* = 1); stiffness, S, (*m* = 2); decrement, D (*m* = 3); mechanical stress relaxation time, R (*m* = 4); and creep, C (*m* = 5).

The oscillation frequency characterizes the tone of a muscle and it is measured as the maximum frequency in the fast Fourier transform spectrum of the accelerogram [33] with a precision of 1.1%. The biomechanical properties are associated with the stiffness and the decrement. Dynamic stiffness, S, characterises the resistance of the muscle to the force that changes its shape. This parameter is calculated as M × a_max_/Δl, where M is the mass of the testing end of the myometer, a_max_ is the maximal acceleration of oscillation, and Δl is the deformation depth of the muscle mass [34]. The reliability/precision of the measurement is 3.9%. The decrement directly measures the dissipation of the oscillation when a tissue recovers the shape after being deformed and is related to muscle elasticity. It is given by D = ln (a_1_/a_3_), where a_1_ and a_3_ are the first two positive amplitudes of the accelerogram. The viscoelastic properties are measured by the relaxation time, R, and the creep, C (known as *number of Deborah*). The relaxation time is the time taken by the muscle to restore its initial shape after external force is removed [35]. It is measured as the time interval between the maximum displacement of the tissue and the return to its initial shape. The reliability of the R measurement is 1.5%. Furthermore, the gradual elongation of a tissue over time when placed under a constant tensile stress is the last extracted quantity, i.e., creep, C, also known as the *Deborah number*; it is measured as the ratio of the relaxation time, R, to deformation time. The device has been used in the multiscan mode, where one measurement corresponded to the mean of 3 mechanical taps [21,36].

Data analysis and visualization have been performed using home-made routines written in MATLAB R2022b under the Windows 11 operating system.

## 4. Results

**Figure 2:**
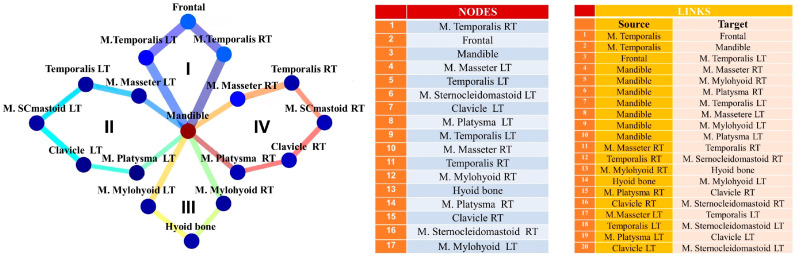
Temporomandibular muscle network. The nodes and links are indicated by full circles and tick lines, respectively. The four cycles are indicated by I, II, III, and IV. The list of nodes and links is also reported. The thickness of the links refers to the measured values of the frequency averaged across all patients for each link (see Figure 3c, top panel). LT (**left**); RT (**right**).

MyotonPRO measurements have been carried out on bone insertion points of the masticatory musculature, forming an osteon–muscular network schematized in Figure 2. This network consists of 17 nodes (full circles). The measurements are performed on the points of contact between two nodes that lie on the links. The network is composed of 20 links (*l* = 1, …, 20) grouped in four cycles (*cy* = 1, …, 4) indicated with I, II, III, and IV, closing on the same mandibular node. The names of nodes and links are tabulated and shown in Figure 2.

### 4.1. Measurement Maps A^m^

**Figure 3:**
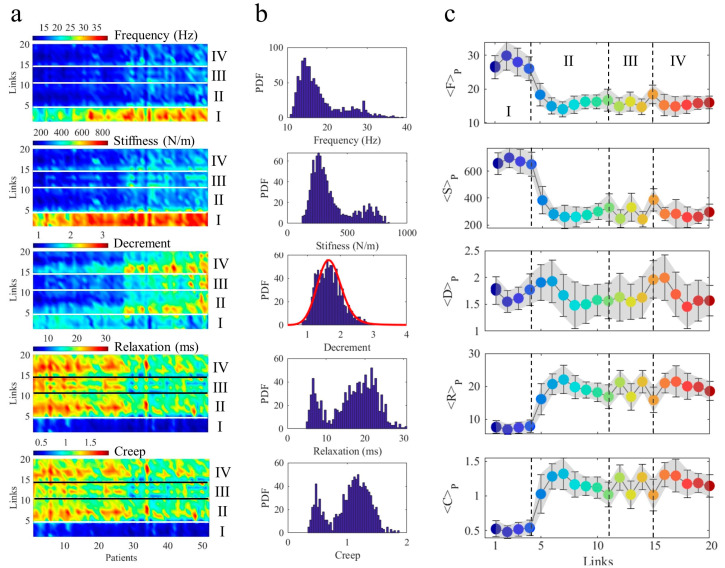
Maps of link measurements. (**a**) Measurements of MyotonPRO in each of the 20 links for each of the 52 healthy patients The horizontal lines delimit the links belonging to cycles I, II, III, and IV. (**b**) Probability distributions of measurement maps showing the bimodal distributions of frequency, stiffness, relaxation, and creep. The bimodal distributions have been modelled as a mixture of two Gaussians whose mean and proportion values of the two components are reported in Table 1. The bimodal distribution is not able to fit decrements in data, modelled by a stable distribution (red line). (**c**) Averaged values of *A^m^_lp_* on all 52 patients, giving <*A^m^_l_*>*_P_* = <*F*>*_P_*, <*S*>*_P_*, <*D*>*_P_*, <*R*>*_P_*, and <*C*>*_P_* (full circles). Each link is represented by a full circle whose colour is the same as the corresponding link in the graph of Figure 2. The standard deviations of <*A^m^_l_*>*_P_* for all links, *l*, are plotted by error bars and represented by shaded areas. PDF (Probability density function); Hz (Hertz); N/m (N/meter); ms (millisecond).

All measured parameters (F, S, D, R, and C) for the *Np* = 52 healthy patients have been visualised by 2D matrices, *A^m^_lp_*, shown as colour maps in Figure 3a. Each pixel (*l*,*p*) corresponds to the measure of the parameter *A^m^* on a specific link, *l*, of a specific patient *p*. The patients have been sorted by age, while links are numbered counterclockwise in Figure 2. The link axis has been grouped by horizontal lines in four different cycles indicated by I, II, III, and IV. The first cycle, I, groups links from *l* = 1…4 and shows higher values for frequency (*m* = 1) and stiffness (*m* = 2) in comparison with all the remaining links. Correspondingly, it assumes smaller values for relaxation time (*m* = 4) and creep (*m* = 5).

Thus, upon this early inspection, the cycle I seems to play a peculiar role in the muscular network. The decrement (*m* = 3) shows a quite different behaviour; indeed, it assumes fluctuating values without any clear dependence on the cycles. To quantify this different behaviour, we have computed the probability density function, PDF, for each map *A^m^*. The results (Figure 3b) show a bimodal distribution for F, S, R, and C (*A^m^* with *m* = 1, 2, 4, and 5, respectively), which is well modelled by a mixture of two Gaussians. The first Gaussian, given by the lower PDF values in F and S, corresponds with F and S values measured on the II, III, and IV cycles, while the second Gaussian corresponds to higher values of F and S measured on the first cycle, I. The opposite behaviour is found for the viscoelastic R and C parameters. The proportion and the mean of the two Gaussian components in the F, S, R, and C measurements are reported in Table 1.

On the other hand, the PDF of decrement cannot be modelled by using a mixture of two Gaussian. It has been fitted by a stable distribution, deviating from Gaussian behaviour and characterised by a longer asymmetric tail. Stable distributions have been applied to several dynamical processes occurring in complex systems with functional disorder characterised by an energy landscape deriving from configurations with nearly degenerate competing levels. These systems constitute a hot topic in the last few decades in several fields, such as biology [37,38], chemistry [39,40] and material science [41,42]. A basic and relevant consequence is the emergence of anomalous phases and unpredictable properties as occurs in quantum matter [43,44,45]. The different distributions of our measurements can also be appreciated by averaging each *A^m^* matrix on all 52 patients for each link, *l*, giving <*A^m^_l_*>*_p_*_=(1,...*Np*)_ = <*F*>*_P_*, <*S*>*_P_*, <*D*>*_P_*, <*R*>*_P_,* and <*C*>*_P_* shown in the five plots of Figure 3c. The larger frequency and stiffness for the first cycle, alongside the larger viscoelastic properties of creep and relaxation time of the II, III, and IV cycles, are well depicted. At the same time, <*D*>*_P_* assumes competing slightly different values in the four cycles. These competing values produce a more complex and dynamic landscape of muscle elasticity in comparison with muscle tone, stiffness, and viscoelasticity.

### 4.2. Cycle Dynamics: Exponential Growth and Decaying of A^m^_cy_ Measurements

**Figure 4:**
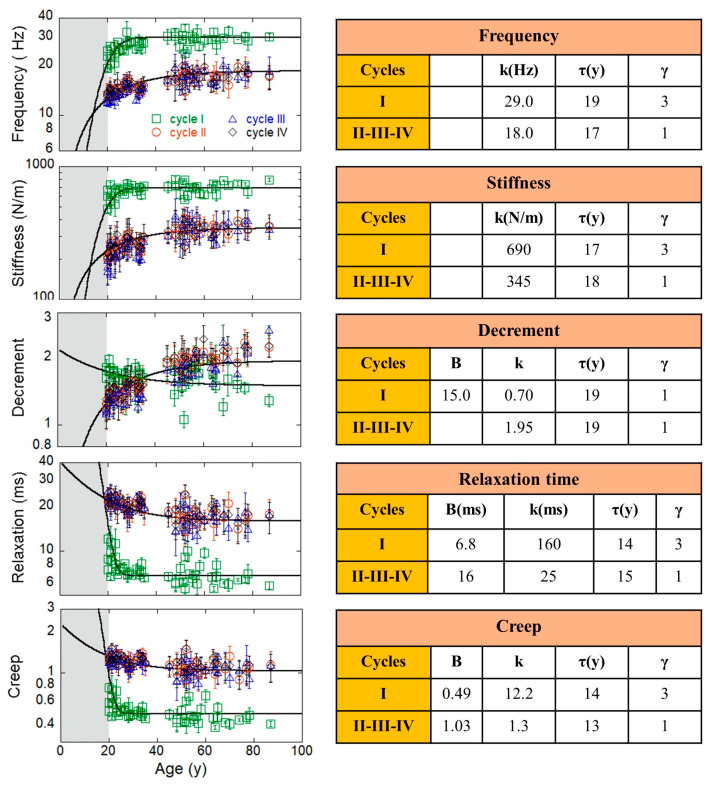
Growing, decaying, and competing cycles in muscle networks. Measurements of MyotonPRO in each cycle as a function of the age of the 52 healthy patients are shown. Values of fit parameters extracted by Equations (1) and (2) are also tabulated. We note that, after 25 years, the parameters’ evolution shows a saturation-like behaviour after τ values. Hz (Hertz); N/m (N/meter); ms (millisecond).

The different values of *A^m^* in links belonging to the different cycles suggest studying the time evolution of the four cycles in our measurements, as shown in the plot of the cycle average *A^m^_cy_* as a function of the age of patients (Figure 3). We observe a stretched exponential growth in *A*^1^*_cy_* = F*_cy_* and *A*^2^*_y_* = S*_cy_*, while the viscoelastic *A*^4^*_cy_* = R*_cy_* and *A*^5^*_cy_* = C*_cy_* show a stretched exponential decay in the same range. We have modelled our data by using the following stretched exponential equations:

(1) $$A_{cy}^{m}(t)=k_{cy}^{m}(1-e^{{-\left(t/\tau_{cy}^{m}\right)}^{\gamma_{cy}^{m}}})$$

where *k^m^_cy_* is a constant, *τ^m^_cy_* is a characteristic time, and *γ^m^_cy_* is the stretching exponent relative to the measurement *m* on the cycle *cy*. Similarly, the stretched exponential decay of viscoelastic quantities is given by

(2) $$A_{cy}^{m}(t)=B_{cy}^{m}+k_{cy}^{m}e^{{-\left(t/\tau_{cy}^{m}\right)}^{\gamma_{cy}^{m}}}$$

where *B^m^_cy_* is the baseline value and *k^m^_cy_* is a constant value. All the parameters, including *B^m^_cy_*, *k^m^_cy_*, *τ^m^_cy_,* and *γ^m^_cy_* for each cycle, *cy*, and each MyotonPRO measured quantity, *m*, are tabulated and shown in Figure 4. The *k^m^_cy_* is larger for the first cycle, indicating that the final value reached with late age is always larger in the first cycle for F*_cy_*(t) and S*_cy_*(t), while it is smaller in the same cycle for the decaying viscoelastic R*_cy_*(t) and C*_cy_*(t).

A different behaviour is observed in the *A*^3^*_cy_* = D*_cy_*(t) evolution. Here, the decrement grows in the first cycle, while it decays in the other cycles. The characteristic time, *τ^m^_cy_*, is around 20 ± 1 years for all cycles in frequency (*m* = 1) and biomechanical muscle evolution (*m* = 2, 3). In the decay of viscoelastic evolution, the characteristic time, *τ^m^_cy_*, assumes lower values of around 13 ± 1 years. The stretching exponent (*γ^m^_cy_*) is 1 for all *A^m^_cy_* in the II, III and IV cycles, while it is 3 for the first cycle (*cy* = 1). Thus, the growth of tone and stiffness, as well as the decay of viscoelastic relaxation time and creep, are faster in the first cycle. In this way, we have well characterised model lines describing the physiological evolution of our system that can serve as references for measurements in non-healthy patients. This will allow for the assessment of disorder degrees at different ages as new diagnostic tools.

### 4.3. Correlation Matrix Analysis of MyotonPRO Measurements

In order to investigate how the complex dynamics of decrement affect the normal behaviour of the other muscle properties, we have performed a correlation study between the *A^m^_lp_* matrices. We first calculated the Pearson correlation coefficients between *A^m^* maps, given by

(3) $$c^{m,m\prime }\;=\;{corr}^{2}(A^{m},\;A^{m\prime })\;=\;\frac{\sum_{l}\sum_{p}\left(A_{lp}^{m}-\bar{A^{m}}\right)\left(A_{lp}^{m\prime }-\bar{A^{m\prime }}\right)}{\sqrt{\left(\sum_{l}\sum_{p}{\left(A_{lp}^{m}-\bar{A^{m}}\right)}^{2}\right)\left(\sum_{l}\sum_{p}{\left(A_{lp}^{m\prime }-\bar{A^{m\prime }}\right)}^{2}\right)}}$$

where *m* and *m*′ = 1, …, 5. Each Pearson correlation coefficient is a measure of the linear association between two variables, *A^m^* and *A^m′^*. It ranges from −1 to 1, where −1 indicates a perfect negative correlation, meaning as one variable increases, the other decreases; a value of 0 indicates no correlation, meaning the variables do not move together; and a value of 1 indicates a perfect positive correlation, meaning as one variable increases, the other also increases. The results are shown in Table 2. Strong positive correlations occur between frequency and stiffness (*C*^23^ = *C*^32^ = 0.96) and between relaxation time and creep (*C*^45^ = *C*^54^ = 0.99). Consequently, frequency and stiffness have high negative correlations with both relaxation time (*C*^14^ = *C*^41^ = −0.93, *C*^24^ = *C*^42^ = −0.95) and creep (*C*^15^ = *C*^51^ = −0.91, *C*^25^ = *C*^52^ = −0.93).

Lower absolute values of correlation coefficients, indicated by the green cells in Table 2, have been found between decrement (*m* = 3) and the other parameters (*m* = 1, 2, 4, 5). Indeed, we find lower positive correlations between decrement and both frequency (*C*^31^ = *C*^13^ = 0.23) and stiffness (*C*^32^ = *C*^23^ = 0.25) as well as higher negative correlations between decrement and both viscoelastic relaxation time (*C*^34^ = *C*^43^ = −028) and creep (*C*^35^ = C^53^ = −0.19).

#### 4.3.1. Pairwise Patient–Patient and Link–Link Correlations of MyotonPRO Measurements A^m^

**Figure 5:**
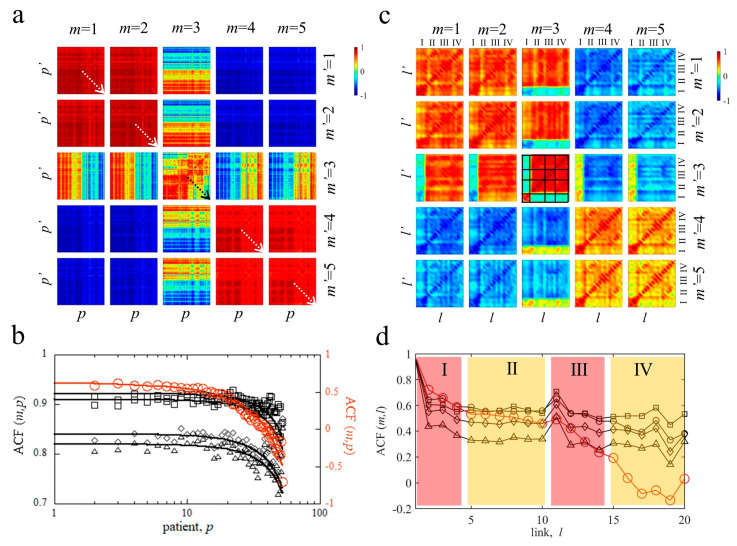
Patient–patient and link–link map correlations in masticatory muscle network. (**a**) Correlation maps, *c^m^*^,*m*′^*_p_*_,*p*′_, where each pixel (*p*,*p*′) is calculated by Equation (4). (**b**) Autocorrelation function, ACF(*m*,*p*), calculated by Equation (5), for each measurement correlation map, *c^m^*^,*m*^*_p_*_,*p*′_. The *ACF* frequency, stiffness, relaxation time, and creep are represented by black squares, circles, diamonds and triangles, respectively; the *ACF* of decrement corresponds to the red circles. The continuous lines in the ACF panel are the best fitted curve obtained by modelling data with the decaying stretched exponential of Equation (6). (**c**) Correlation maps, *c^m^*^,*m*′^*_l_*_,*l*′_, where each pixel (l,l’) is calculated by Equation (7). The zones delimited by the black thick lines represent cycles I, II, III, and IV. (**d**) Autocorrelation function, *ACF*(*m*,*l*), calculated by Equations (4) and (5), respectively, for the symmetric correlation maps, *c^m^*^,*m*^*_l_*_,*l*′_. ACF (AutoCorrelation Function).

The different distribution of decrement values across the different cycles of the network produces lower correlations between decrement and the other measured parameters. To deepen this aspect, we have calculated the Pearson pairwise correlation coefficients between each pair of patients (p, p′) in the different MyotonPRO maps, *A^m^* (*m* = 1, 2, 4, 5,). As a result, we obtain patient–patient cross-correlation matrices, *c^m^*^,*m′*^*_p_*_,*p′*_:

(4) $${c^{m,m\prime }}_{p,p\prime }\;=\;corr({A^{m}}_{lp},\;{A^{m\prime }}_{lp\prime })\;=\;\frac{\sum_{l=1}^{Nl}\left(A_{lp}^{m}-\bar{A_{p}^{m}}\right)\left(A_{p^{\prime }l}^{m^{\prime }}-\bar{A_{{lp}^{\prime }}^{m^{\prime }}}\right)}{\sqrt{\sum_{l=1}^{Nl}{\left(A_{lp}^{m}-\bar{A_{p}^{m}}\right)}^{2}\sum_{l^{\prime }=1}^{Nl}{\left(A_{l^{\prime }p^{\prime }}^{m^{\prime }}-\bar{A_{p^{\prime }}^{m^{\prime }}}\right)}^{2}}}$$

where Alm¯=∑p=1NpAlpmNp, *m*, and *m*′ = 1, …, 5, and *p* = 1, …, *Np*. The colour cross-correlation maps in Figure 5a highlight, once again, the different dynamics of the decrement. Indeed, while the cross correlation maps with *m*, *m*′ = 1,2,4,5 appear quite homogeneous, the correlation maps involving the decrement with *m* or *m*′ equal to 3 show patterns characterised by stronger discontinuities. When *m* = *m*′, *c^m^*^,*m*^*_p_*_,*p*′_ is a symmetric matrix made by the pairwise linear correlation coefficient between the same measured parameter, *m*, for different patients, *p* and *p*′. Its main diagonal, where *p* = *p*′, is thus composed of *Np* elements equal to 1 since it represents the correlation of a measurement of a parameter for a patient with the same parameter measured in the same patient. As one gets away far from this diagonal, following the dashed arrows in the symmetric maps of Figure 4a, the mean of the diagonal elements is expected to change for dynamic systems. Thus, to describe analytically how each symmetric map, *c^m^*^,*m*^*_p_*_,*p*′_, changes, we have calculated the 1D autocorrelation function, *ACF*, by the mean values of the diagonals of each *c^m^*^,*m*^*_p_*_,*p*′_ matrix:

(5) $$ACF\left(m,p\right)={\left\{\frac{1}{Np-j}\sum_{i=1}^{Np-j}C^{m}\left(p_{i},p_{i+j}\right)\right\}}_{j=0,\ldots ,Np-1}$$

We can now observe that its behaviour decreases for successive diagonals running, as indicated by the dashed arrows in the symmetric maps of Figure 4a, corresponding to the increasing age of the patient. The ACFs for each *c^m^*^,*m*^*_p_*_,*p*′_ map are shown by different symbols in the upper panel of Figure 5b. The specific decay of the ACF is used to quantify the specific dynamic of the process. Also, in this case, we have modelled the ACF’s decay by using the stretched exponential function:

(6) $$ACF\left(m,p\right)=b+\left(a-b\right)e^{{-\left(p/p_{0}\right)}^{\beta }}$$

Here, the decay occurs as a function of the number, *p*, of patients, corresponding with their age; thus, *p*_0_ is related to a characteristic age for the correlation decay of the measured parameter. The stretching exponent, *β*, is a shape parameter characterizing the degree of deviation from an exponential function and the fastness of the decay, *b* is the baseline, and (*a* − *b*) is defined as the contrast and indicates the strength of the decay. These parameters, for all measurements, *m*, are reported in Table 3, while the best fitted curves are shown by the thick lines in Figure 5b. We note how decrement correlation decay is stronger since its contrast, *a* − *b*, is larger; at the same time, it has a stretching exponent near to 1, lower than 1.8, found for the decay of the other parameters. Thus, the decrement correlation decay is stronger and slower. Finally, we observe that the characteristic age for the correlation decrement decay is 50 years, which is lower than the characteristic age of the other parameters’ correlation decay. Indeed, patient–patient correlations decay at 60 years in frequency and stiffness, while decay between these correlations occurs at 70 years in viscoelastic creep and relaxation time.

Now, we move to study the different dynamics of decrement by cross-correlation matrices, *c^m^*^,*m*′^*_l_*_,*l*′_, calculated for all healthy patients, *p*, in each pair of links, *l*, *l*′, as follows:

(7) $${c^{m,m\prime }}_{l,l\prime }\;=\;corr({A^{m}}_{lp},\;{A^{m\prime }}_{l\prime p})\;=\;\frac{\sum_{p=1}^{Np}\left(A_{lp}^{m}-\bar{A_{l}^{m}}\right)\left(A_{l^{\prime }p}^{m^{\prime }}-\bar{A_{l^{\prime }}^{m^{\prime }}}\right)}{\sqrt{\sum_{p=1}^{Np}{\left(A_{lp}^{m}-\bar{A_{l}^{m}}\right)}^{2}\sum_{p^{\prime }=1}^{Np}{\left(A_{l^{\prime }p^{\prime }}^{m^{\prime }}-\bar{A_{l^{\prime }}^{m^{\prime }}}\right)}^{2}}}$$

where Apm¯=∑l=1NlAlpmNl, *m*, *m*′ = 1, …, 5, and *l*, *l*′ = 1, …, *Nl*.

The *c^m^*^,*m*′^*_l_*_,*l*′_ maps shown in Figure 5c give, in this case, the pairwise linear cross-correlation coefficients between link *l* in *A^m^_lp_* matrix and link *l*′ in *A^m’^_l’p_*. We observe that this link–link correlation decay is quite different from the decay in the patient–patient correlation map, *c^m^*^,*m*′^*_p_*_,*p*′_*,* described in Figure 5b. In this case, the 1D autocorrelation function is given by

(8) $$ACF\left(m,l\right)={\left\{\frac{1}{Nl-j}\sum_{i=1}^{Nl-j}C^{m}\left(l_{i},l_{i+j}\right)\right\}}_{j=0,\ldots ,Nl-1}$$

It shows quite different line shapes (see Figure 5d). In particular, the decrement ACF (full red circles) shows a stronger and faster decay. Furthermore, we observe that in the link–link cross correlation maps between decrement and the other quantities, the links belonging to cycle I show an opposite tendency in comparison with the other remaining cycles II, III, and IV, where we receive negative correlations *c*^1,3^*_l_*_,{1,..,4}_, *c*^2,3^*_l_*_, {1,..,4}_, *c*^4,3^*_l_*_, {5,..,20}_, and *c*^5,3^*_l_*_, {5,..,20}_; on the opposite side, *c*^1,3^*_l_*_, {5,..,20}_, *c*^2,3^*_l_*_, {5,..,20}_, *c*^4,3^*_l_*_, {1,..,4}_, and *c*^5,3^*_l_*_, {1,..,4}_ are positive. The decrement symmetric matrix presents negative correlations *c*^3,3^_{1,..,4},{5,..,20}_ and *c*^3,3^_{5,..,20},{1,..,4}_. Thus, also from the above correlation analysis, a peculiar role of cycle I in the muscular network is apparent.

#### 4.3.2. Correlations between Network Cycles of MyotonPRO Measurements

To further quantify the prominent role and the different dynamics of the first cycle, we have first averaged the links in the measurement maps, *A^m^*, for each patient in the four cycles I, II, III, and IV (*cy* = 1, 2, 3, 4) and then we have extracted *i*) the Pearson coefficient *C^m^_cy_*_,*cy*′_ between *A^m^_cy_* and *A^m^_cy′_* matrices and *ii*) the Pearson coefficient *C^cy^_m_*_,*m*′_ between *A^m^_cy_* and *A^m′^_cy_* matrices:

*C^m^_cy,cy_*_′_= *corr*^2^ (*A^m^_cy_*, *A^m^_cy_*_′_) (9)

*C^cy^_m,m_*_′_= *corr*^2^ (*A^m^_cy_*, *A^m′^_cy_*) (10)

**Figure 6:**
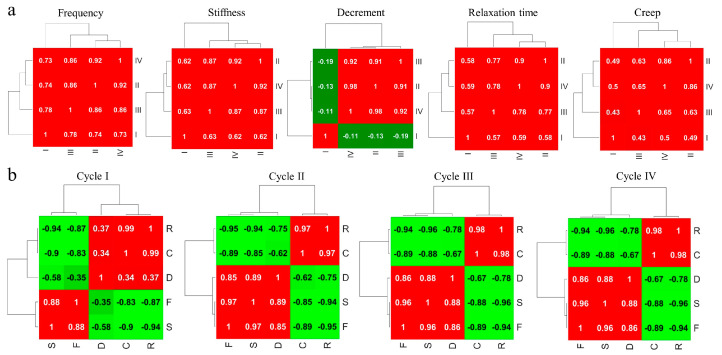
Heat map with dendrogram of coefficients (**a**) *C^m^_cy_*_,*cy*′_, calculated by Equation (8) and (**b**) *C^cy^_m_*_,*m*’_ calculated by Equation (9). The Pearson coefficients are reported also reported. F (frequency); S (stiffness); D (decrement); C (creep); R (relaxation).

Coefficients *C^m^_cy_*_,*cy*′_ tell us how a measurement of a cycle is correlated with another cycle in the same measurement *A^m^*. Coefficients *C^cy^_m m_*_′_ describe how a measurement, *m*, of a cycle is correlated with another measurement, *m*′, for the same cycle. The results are shown by clustergrams in Figure 6. A clustergram is composed of a heat map of the correlation matrix (*C^m^_cy_*_,*cy*′_ and *C^cy^_m_*_,*m*′_, where the rows and columns are sorted in the order suggested by the hierarchical clustering). This allows for the grouping of various subsets of the cycles that are highly correlated within the subset, as highlighted by a dendogram.

*C^m^_cy_*_,*cy*′_, as shown in Figure 6a, has a positive result, indicating positive correlations between *A^m^* measurements in all *cy* cycles, except for the decrement (*m* = 3), where the first cycle is negatively correlated with the other cycles. Stronger positive correlations occur between the lateral cycles in creep, where *c^m^*_24_ = *c^m^*_42_ >0.86. *C^cy^_m m_*_′_ are shown in Figure 6b. Equal *dendrograms* with similar heat maps are found for cycles II, III, and IV. Here, *C^cy^_m m_*_′_ is positive between F, S, and D (*m*,*m*′ = 1, 2, 3) and between R and C (*m*,*m*′ = 4, 5), while it is negative between R and C and F, S, and D. This clustering changes in cycle I, where negative correlations occur between F and D.

## 5. Discussion

We have studied the masticatory system in its functional state by using myotonic measurements on specific points drawing a muscular network. This network is composed of 17 nodes and 20 links grouped in four cycles closing on the mandibular node. Myotonic measured parameters are frequency, F, stiffness, S, decrement, D, relaxation time, R, and creep, C. They characterise the tone (F) as well as the biomechanical (S, D) and viscoelastic (R and C) properties of the system. The state of the system has been represented as a matrix, *A^m^*, (visualised as a colour map) for each measured parameter, *m* = *F*, *S*, *D*, *R*, *C*, where a specific element *A^m^_lp_* provides the measured parameter of the link, *l*, in the patient, *p*. In this way, statistical physics has been used to characterise the functional state of the system and to describe its evolution with age. All measured maps have shown a bimodal distribution due to the different values measured for the first cycle, I, with respect to the other (II, III, and IV) cycles, except for the decrement, which assumes nearly competing values on all links, giving rise to a stable distribution typical of nonlinear and metastable phenomena. Indeed, the decrement (inversely proportional to the elasticity) seems to be a critical parameter of the masticatory musculature, while the first cycle might play a prominent role in its functionality. The time evolution analysis of our data clustered in the four cycles shows a clear stretched exponential growth of F and S for all cycles, but the first cycle shows a faster growth. Similarly, we find a decay of C and R for all cycles, with a faster decay in the first cycle. The decrement behaviour is quite different, confirming the critical nature of elasticity in the functionality of the masticatory system. Indeed, it decays in the first cycle, while, at the same time, it increases for the other (II, III, and IV) cycles at the same rate.

The peculiar role of cycle I and the critical behaviour of decrement comes clearly from the matrix cross-correlation analysis. While tone and stiffness, as well as relaxation and creep, are highly correlated, the decrement shows lower correlations, positive with F and S and negative with C and R. The pairwise patient–patient correlations appear quite homogeneous between F and S as well as between C and R, while, in the case of decrement, a non-homogeneous pattern arises. The pairwise cross-correlation homogeneity and their forming pattern are related to the dynamics of the system. This dynamic has been characterised by calculating the 1D autocorrelation function, ACF, indicating a stronger decay dynamic of correlations for the decrement. Furthermore, this decay occurs earlier for the decrement, around the 50th year, while the decay characteristic time of the biomechanical properties F and S is around the 60th year and the viscoelastic R and C decay occurs later towards the 70th year. The partial link–link correlation matrices confirm the stronger decay of the 1D autocorrelation function of the decrement and the peculiar behaviour of the links belonging to the first cycle.

## 6. Conclusions

In summary, we have defined a quantitative evolutive trend of the functional TMJ system based on statistical–physics modelling of myotonic measurements performed on 52 healthy patients. Our modelling highlights the importance of the first cycle in the functionality of the masticatory system and the critical nature of the decrement parameter. Additionally, it provides an understanding of the system’s dynamics and the correlations between the different measured parameters. This model could offer deeper insights into the understanding and classification of temporomandibular disorders by investigating the extent of deviation from the physiological trend in measurements performed in non-healthy patients. Indeed, the taxonomy of the temporomandibular joint (TMJ) is primarily based on clinical observations, patient symptoms, and expert consensus [29,46]. Thus, a significant gap exists in this taxonomy due to the absence of a physical–mathematical model. Despite the complexity of TMJ disorders, involving biological, psychological, and social factors, we believe that methodologies based on quantitative models could significantly enhance the current taxonomy and contribute positively to this field, improving the diagnosis and treatment of muscular disorders in the masticatory region.

## Figures and Tables

**Table 1:** Proportion and mean of the two Gaussian components used to fit the probability density function (PDF) of *A^m^* with *m* = 1, 2, 4, 5. F (frequency); S (stiffness); R (relaxation); C (creep).

Cycles	F (%)	S (%)	R (%)	C (%)	<F>	<S>	<R>	<C>
I	0.32	0.27	0.20	0.20	25.50	636.0	7.17	0.49
II–IV	0.68	0.73	0.80	0.80	15.35	284.4	18.90	1.16

**Table 2:** Correlation coefficients, *C^m^*^,*m*′^, between *A^m^* maps calculated by Equation (1). The green cells highlight the different lower values of the *C*^3,*m*′^ coefficients involving decrement.

	Frequency	Stiffness	Decrement	Relaxation	Creep
Frequency	1	0.96	0.23	−0.93	−0.91
Stiffness	0.96	1	0.25	−0.95	−0.93
Decrement	0.23	0.25	1	−0.28	−0.19
Relaxation	−0.93	−0.95	−0.28	1	0.99
Creep	−0.91	−0.93	−0.19	0.99	1

**Table 3:** Fit parameters of *ACF*(*m*,*p*) modelled by Equation (6).

M	Measurement	Contrast	β	p_0_	Age (y)
1	Frequency	0.028	1.85	42	60
2	Stiffness	0.028	1.85	43	60
3	Decrement	0.265	1.05	33	50
4	Relaxation	0.050	1.85	48	70
5	Creep	0.040	1.85	48	70

## Data Availability

Data sharing is not applicable to this article.
